# Climate change amplifies global mycotoxin exposure: Emerging risks and sustainable solutions

**DOI:** 10.1002/imo2.70131

**Published:** 2026-08-06

**Authors:** Mengyao Xue, Zheng Qu, Tingting Jiang, Antonio F. Logrieco, Antonio Moretti, Ke Dong, Yanpo Yao, Qi Zhang, Peiwu Li

**Affiliations:** ^1^ Agro‐Environmental Protection Institute, Ministry of Agriculture and Rural Affairs Tianjin China; ^2^ Xianghu Lab, Biomanufacturing Institute Hangzhou China; ^3^ Institute of Sciences of Food Production, National Research Council Bari Italy; ^4^ Kyonggi University Seoul South Korea; ^5^ Key Laboratory of Detection for Mycotoxins, Ministry of Agriculture and Rural Affairs, Oil Crops Research Institute, Chinese Academy of Agricultural Sciences Wuhan China

## Abstract

Climate change intensifies mycotoxin contamination through rising temperatures, elevated CO_2_, and altered precipitation. Global surveys reveal a high and widespread prevalence of major mycotoxins in staple cereals, with low‐income countries bearing the greatest burden. This escalating contamination poses a growing threat to global food security, compromising both food availability and safety. To mitigate these risks, we propose four actions: establish a predictive global surveillance system integrating climate‐crop‐fungal models, deploy field‐ready biosensors, scale up climate‐resilient agriculture and resistant varieties, and implement a multi‐stakeholder governance protocol focused on developing regions. These measures offer feasible solutions to protect global food security in a changing climate.

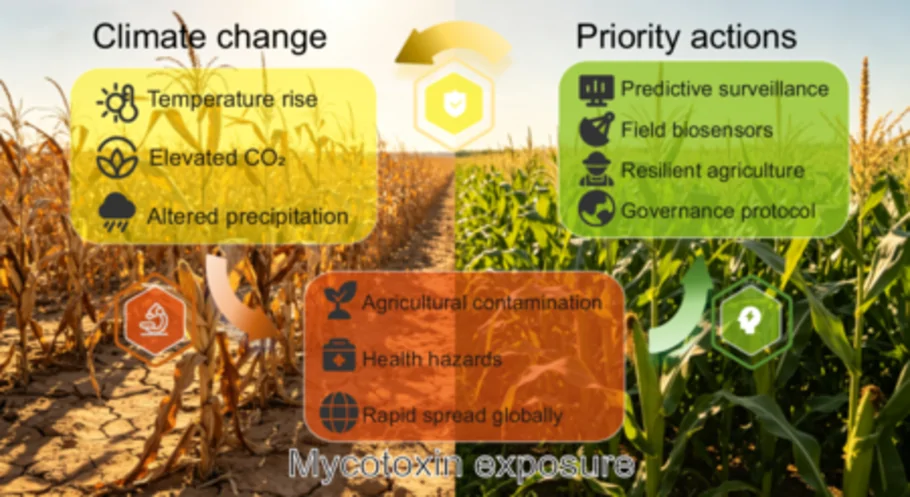

The achievement of food security, as outlined in the United Nations Sustainable Development Goal 2, is increasingly threatened by mycotoxin contamination exacerbated by climate change. Rising temperatures have driven poleward migration of pathogenic fungi at approximately 2.7 km/year since the 1960s. Elevated CO_2_ alters plant physiology and directly modulates fungal growth and toxin biosynthesis. Changing precipitation patterns, including droughts, floods, and humidity fluctuations, further shape region‐specific contamination risks. Despite the complex interplay of the factors, current predictive models remain inadequate, and low‐income countries face disproportionate vulnerability due to limited infrastructure and technical capacity. The commentary proposes four priority actions: the development of a predictive global surveillance system, deployment of field‐based biosensors, climate‐resilient agriculture with resistant varieties, and establishment of a multi‐stakeholder risk management framework prioritizing capacity‐building in developing regions. Collectively, these present feasible approaches to safeguarding global food security and public health amid a changing climate.

Mycotoxins are toxic secondary metabolites naturally produced by fungal species, such as *Aspergillus* and *Fusarium*. The compounds commonly contaminate food supplies, thereby posing a significant risk to food safety and public health. Aflatoxins (AFs) and deoxynivalenol (DON) are commonly found in wheat, maize, and barley, while fumonisins (FBs) predominantly contaminate maize and wheat [1]. Ochratoxin A (OTA) is frequently present in a variety of foodstuffs and crops, including coffee, beer, and vegetables [2]. According to a global ten‐year survey (2008–2017) from 100 countries, the prevalence of major mycotoxins in cereals was 64% for DON, 60% for FBs, 45% for zearalenone (ZEN), 23% for aflatoxin B1 (AFB1), 19% for T‐2 toxin, and 15% for OTA [1]. Over the same period, AFs were detected in nearly all cereal grains, with a prevalence of 100% in maize. The World Health Organization has classified AFB1 as a Group I carcinogen, estimated to contribute to approximately 25,200–155,000 cases of hepatocellular carcinoma annually worldwide. A more recent 2025 global survey by Cargill, encompassing 151,895 samples across 41 countries, further confirmed the widespread occurrence of mycotoxins, with contamination rates above detection limits reaching 78% for DON, 77% for FBs, 72% for ZEN, 69% for OTA, and 64% for AFs [3]. Notably, 34% of all samples tested exceeded performance risk thresholds associated with at least 0.5% production loss in livestock.

Over the past century, the rate of carbon dioxide emissions into the atmosphere has contributed to an increase in the Earth's average surface temperature by approximately 1.18°C. The climatic changes directly influence mycotoxin contamination patterns through multiple mechanisms. Rising temperatures expand the geographical range of toxigenic fungi, driving a poleward shift of pathogen distribution at an average rate of approximately 2.7 km per year since the 1960s [4]. Simultaneously, elevated temperature and water activity levels modulate fungal growth and mycotoxin biosynthesis, with different mycotoxins showing distinct optimal conditions for production (Table S1). As a result, contamination patterns are shifting geographically and quantitatively. Scientific evidence indicates that global warming is expected to continue, with projected temperature increases ranging from 1.4°C to 5.5°C over the next 10 decades [5]. Consequently, climatic temperature plays a critical role in the infection of crops by mycotoxigenic fungi, potentially facilitating a more rapid global dissemination of mycotoxins. Meanwhile, elevated atmospheric CO_2_ levels could also influence mycotoxin contamination through multiple mechanisms. Rising CO_2_ concentrations alter plant physiology, including changes in carbon–nitrogen balance and defense signaling pathways, which may increase host susceptibility to fungal infection. Additionally, elevated CO_2_ has been shown to directly modulate fungal growth and mycotoxin biosynthesis in several toxigenic species, though the effects are species‐ and toxin‐specific [6]. These CO_2_‐driven changes interact with temperature and humidity to shape overall contamination risk.

From a geographical standpoint, the greatest risk of mycotoxin contamination driven by climate change is anticipated in developing nations characterized by temperate climates. Specific mycotoxins are projected to increase in prevalence under conditions of elevated temperatures combined with high humidity. In Southeast Asia, rising temperatures coupled with monsoonal rainfall are likely to exacerbate AF contamination in maize. In sub‐Saharan Africa, severe drought conditions induced by higher temperatures, alongside inadequate storage practices, have substantially amplified the risk of FBs contamination in maize and OTA contamination in wheat [7].

Global climate warming exerts a substantial impact on the distribution of mycotoxin contamination. Attaining a thorough understanding of the precise effects that future climate change will have on mycotoxin concentrations remains a significant challenge. The opinion aims to outline the present status and potential transmission risks of global mycotoxin pollution within the framework of climate warming, and to provide focused recommendations for managing its emerging trends.

## 1 MYCOTOXINS EXERT SIGNIFICANT EFFECTS ON HUMAN HEALTH

To date, researchers have identified and characterized more than 400 mycotoxins [6]. The physicochemical characteristics of mycotoxins, especially their hydrophilic‐hydrophobic equilibrium, play a crucial role in determining their environmental behavior and transfer within the food chain. Hydrophobic mycotoxins, preferentially partition into lipid‐rich substrates, resulting in their accumulation in lipid‐rich animal products such as milk, eggs, and fatty tissues [8]. Conversely, mycotoxins with greater hydrophilicity predominantly remain in aqueous environments, influencing their persistence in plant tissues and their potential for subsequent transfer into animal products [9]. Numerous mycotoxins have been linked to carcinogenic, mutagenic, reprotoxic, and immunotoxic effects, in addition to causing toxicity in the liver and kidneys. The most commonly observed outcome of acute mycotoxin exposure is impaired liver or kidney function, which can be fatal in severe cases (Figure 1).

**Figure 1:**
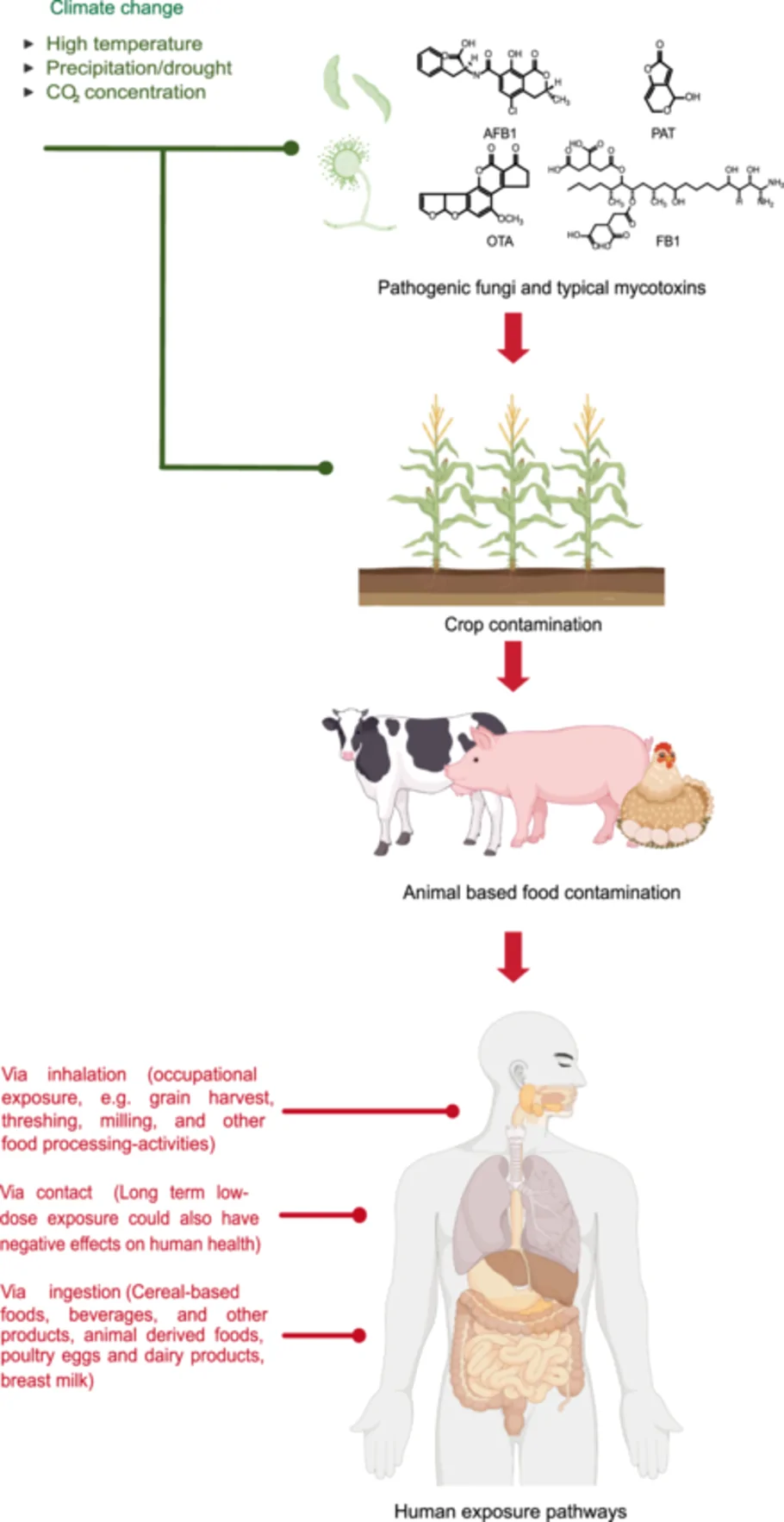
Schematic overview of climate‐driven mycotoxin contamination and human exposure pathways. Rising temperature, CO_2_, and humidity promote fungal growth and mycotoxin biosynthesis. Humans are exposed via inhalation, dermal contact, and ingestion of contaminated crops and derived foods. The image materials used in Figure 1 are provided by Figdraw.

Empirical evidence indicates that certain populations are disproportionately vulnerable to the adverse effects of mycotoxins. Multiple studies have documented a high prevalence of exposure to various mycotoxins among young children. A meta‐analysis by Danesh‐Maskooni et al. reported that AFB1 exposure was significantly associated with elevated risks of stunting and underweight in infants and children, with pooled analysis showing negative associations with height‐for‐age and weight‐for‐age z‐scores [10]. In a UK biomonitoring study of children aged 2.4–6.8 years, DON was detected in 95.6% of urine samples, and 66% of children showed co‐exposure to three or more mycotoxins, with 34.2% of samples exceeding the tolerable daily intake for DON [11]. Similarly, a Brazilian study reported that 63% of children and adolescents (aged 3–17 years) exhibited co‐exposure to 2–6 mycotoxins, with hazard quotient values exceeding 1 for OTA in 100% of preschoolers [12]. These toxins have been implicated in a range of detrimental growth outcomes in infants and young children. Notably, some mycotoxins have been reported that they possess the ability to cross the placental barrier as well as the blood‐brain barrier, thereby posing significant health risks to both fetuses and pregnant individuals [13]. Furthermore, occupational exposure to mycotoxins is a concern for workers in agricultural settings, warehouses, and food and feed processing facilities. Work environments characterized by poor ventilation, inadequate protective clothing, and insufficient safety equipment exacerbate the risk of mycotoxin contamination among these workers.

## 2 THE GLOBAL INCIDENCE OF MYCOTOXIN CONTAMINATION CONTINUES TO RISE IN A CHANGING CLIMATE

Currently, various climate‐related factors increasingly jeopardize global food security by influencing mycotoxin contamination through multiple mechanisms. Elevated temperatures might directly stimulate fungal growth and augment mycotoxin biosynthesis, while also enabling the geographic expansion of toxigenic species [14]. Since the 1960s, climate change has induced a poleward migration of pathogens at an average rate of approximately 2.7 ± 0.8 km per year, thereby extending contamination into higher‐latitude regions [15]. Under environmental selection pressures, rising temperatures further facilitate the emergence of novel pathogens and intensify the virulence of established toxigenic fungi, particularly enhancing their toxin‐producing capabilities [14]. Additionally, warmer and more humid conditions alter the seasonal phenology of pathogens, accelerating mold reproduction rates [15]. Variations in precipitation patterns, including drought, flooding, and humidity fluctuations, affect fungal colonization and toxin production in region‐specific manners [16, 17]. Elevated atmospheric CO_2_ concentrations also impact plant physiological processes and fungal metabolism, with effects that differ according to species and toxin type [6].

Over the past decades, researchers have issued progressively more frequent warnings regarding global warming, associating it with the proliferation of mycotoxin‐producing fungi in diverse geographic regions worldwide. Recent monitoring efforts have documented a continued high prevalence of mycotoxins across major grain‐producing regions, with contamination rates above detection limits reaching over 75% for DON, and over 70% for FBs and ZEN globally [3, 18]. Further analyses using the AFLA‐maize mechanistic model demonstrate that in maize cultivation areas with mean annual temperatures ranging from 15°C to 32°C, each 1°C–2°C increase correlates with an 8.3% rise in the probability of AFB1 contamination exceeding the EU regulatory limit (5 μg/kg) at harvest [19]. In the context of a changing climate, mycotoxin contamination is anticipated to increase by 30% to 180% across various countries and regions in 30 years (Figure 2A). In southeastern Africa, the incidence of mycotoxin contamination in maize and rice is projected to potentially double by 2050 under the representative concentration pathway 8.5 scenario. In Eastern Europe, warmer winter conditions are projected to enhance the overwintering survival of pathogens, thereby increasing the risk of DON contamination in maize and wheat (Figure 2B–G). Compared with middle and high‐income countries, low‐income countries are more severely affected by climate change and fungal toxin pollution [20]. As reported in the FAO 2023 Annual Report, the incidence of mycotoxin exceedance in cereals within low‐income countries rose from 14% to 19% between 2018 and 2022 (http://www.fao.org/food-safety/en/). The trend is expected to be particularly significant in sub‐Saharan Africa, where additional challenges such as inadequate storage infrastructure and limited monitoring capabilities persist. These emerging challenges highlight the critical necessity for effective strategies to mitigate the spread of mycotoxin contamination amid ongoing climatic changes.

**Figure 2:**
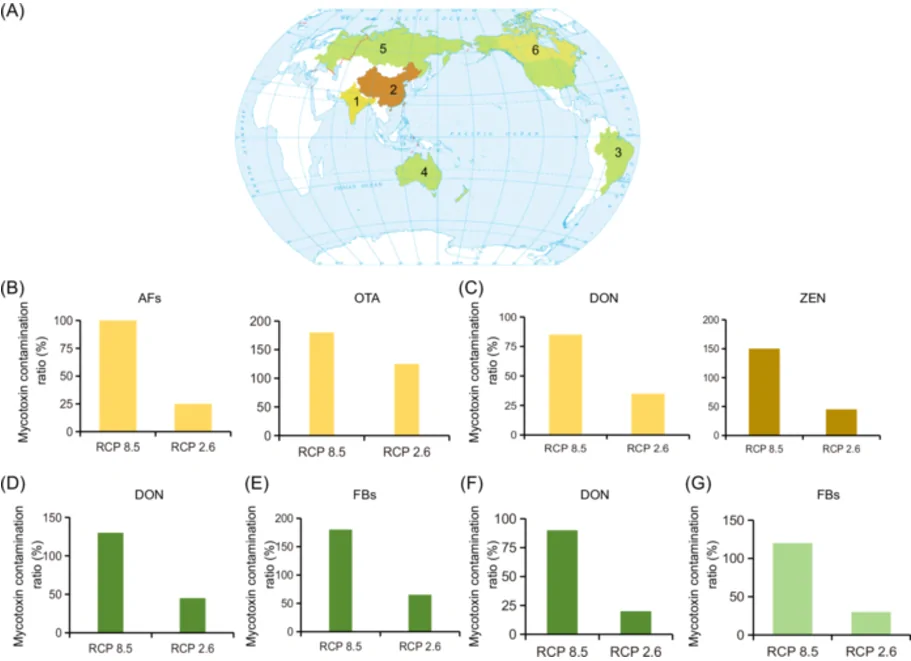
Predicted changes in the infection risk of mycotoxins under climate change scenarios. (A) Reported regions with elevated risks of mycotoxin infections in the future. (1) South Asia represented by India and Bangladesh; (2) East Asia represented by China; (3) South America represented by Brazil; (4) Oceania countries represented by Australia and New Zealand; (5) Eastern Europe represented by Russia and Ukraine; (6) North America represented by Canada. (B) South Asia (exemplified by India and Bangladesh). (C) East Asia (exemplified by China). (D) South America (exemplified by Brazil). (E) Oceania (exemplified by Australia and New Zealand). (F) Eastern Europe (exemplified by Russia and Ukraine). (G) North America (exemplified by Canada). The base world map in (A) was obtained from the Standard Map Service System (http://bzdt.ch.mnr.gov.cn/index.html). Drawing review number: No. GS(2016)2957. (B–G) show the predicted relative changes (%) in contamination ratio for mycotoxin infection risk under different representative concentration pathways (RCP 8.5 and RCP 2.6). AFs: aflatoxins Total; OTA: ochratoxin A; DON: deoxynivalenol; FBs: fumonisins total; ZEN: zearalenone; RCP 8.5: Rising radiative forcing pathway leading to 8.5 W/m^2^ (~1370 ppm CO_2_ eq) by 2100; RCP 2.6: Peak in radiative forcing at ~3 W/m^2^ (~490 ppm CO_2_ eq) before 2100 and then decline (the selected pathway declines to 2.6 W/m^2^ by 2100).

## 3 SUGGESTIONS AND MEASURES TO ADDRESS THE SPREAD OF MYCOTOXINS

Owing to the intricate nature of climatic variables, robust simulation studies addressing mycotoxin contamination remain insufficient, and there are still many unknowns regarding the mechanisms by which high temperature conditions lead to an increase in fungal toxin contamination. We hereby propose the following suggestions (Figure S1).

Firstly, we prioritize the development of a global surveillance network that integrates climate, crop, and fungal growth models into a multi‐scale predictive framework. The system should focus on high‐latitude regions, where mycotoxin contamination is increasingly observed but predictive capacity remains inadequate, and provide timely, region‐specific risk forecasts to guide preemptive interventions.

Secondly, it is essential for the development and field deployment of advanced biosensing technologies for mycotoxin detection. As summarized in Table S2, antibody‐based and DNA receptor‐based biosensors are the most promising, offering rapid, in‐situ, and accurate detection of both toxigenic fungi and mycotoxins [10]. However, their widespread application is currently hindered by high production costs, insufficient field stability, and a lack of validation across diverse crop matrices. We therefore call for targeted research investment to overcome these barriers and to prioritize regulatory pathways that enable real‐time mycotoxin early warning systems.

Thirdly, source‐level prevention comprising two complementary actions is essential and necessary. One, systematically promote climate‐resilient agricultural practices to suppress fungal proliferation and toxin production by improving soil health and optimizing field microclimates. The other is to accelerate breeding of climate‐adapted, pathogen‐resistant crop varieties tailored to local climate projections. These measures, combined with targeted use of biological and chemical agents, represent the most effective and sustainable first‐line defense against pre‐harvest mycotoxin contamination.

Last, we consider the establishment of a global mycotoxin risk management protocol supported by a multi‐stakeholder collaborative governance mechanism. Immediate capacity‐building efforts should focus on developing countries, where insufficient monitoring infrastructure, limited technical capacity, and low public awareness create the greatest vulnerability. Enhanced international cooperation is essential to systematically integrate technological, policy, and local resources for effective mycotoxin containment worldwide.

In summary, addressing the intersection between climate change and mycotoxin contamination has evolved from a specialized scientific issue to a critical element in securing global food security. We prioritize four actions to mitigate climate‐driven mycotoxin risks: global predictive surveillance, field‐deployable biosensors, climate‐resilient agriculture and resistant varieties, and a governance protocol focused on developing countries. These actions represent the most feasible pathways to safeguard global food security and public health in a warming world.

## AUTHOR CONTRIBUTIONS

Yanpo Yao, Qi Zhang, and Peiwu Li provided direction and guidance throughout the preparation of this manuscript. Mengyao Xue and Zheng Qu wrote and edited the manuscript. Mengyao Xue generated and revised the figures. Tingting Jiang, Antonio F. Logrieco, Antonio Moretti, and Ke Dong revised the manuscript. All authors have read the final manuscript and approved it for publication.

## CONFLICT OF INTEREST STATEMENT

The authors declare no conflicts of interest.

## ETHICS STATEMENT

No animals or humans were involved in this study.

## AI STATEMENT

During the preparation of this manuscript, the authors used DeepSeek‐V3 (large language model developed by DeepSeek Company) for language refinement and grammar correction. After using this tool, the authors reviewed and edited all AI‐generated suggestions as needed and took full responsibility for the final content of the publication.

## Supporting information


**Figure S1:** Schematic overview of the four priority actions proposed to mitigate climate‐driven mycotoxin contamination.

**Table S1:** The optimal temperature and water activity of mycotoxin production.
**Table S2**: Comparison of major mycotoxin detection methods.

## Data Availability

The data that support the findings of this study are available from the corresponding author upon reasonable request. The data and material related to mycotoxin levels and meteorological models generated by the Agro‐Environmental Protection Institute, Ministry of Agriculture and Rural Affairs, are not publicly available due to legal, institutional, and confidentiality restrictions. Requests for access to the datasets should be directed to the corresponding authors: Yanpo Yao (yaoyanpo@caas.cn), Qi Zhang (zhangqi01@caas.cn), and Peiwu Li (lipeiwu@caas.cn). Approved applicants will be required to sign a data access agreement, which includes obligations regarding third‐party data sharing and the protection of participant privacy. Users are also required to appropriately acknowledge the source of the data in any publications or presentations arising from their use. Supplementary materials (methods, figures, tables, graphical abstract, slides, videos, Chinese translated version, and updated materials) could be found in the online DOI or iMetaOmics http://www.imeta.science/imetaomics/.
