# Supplementary material for: Climate change amplifies global mycotoxin exposure: Emerging risks and sustainable solutions
Source: IMetaOmics. 2026 Aug 6;3(3):e70131. doi: 10.1002/imo2.70131 (PMC13674422; doi:10.1002/imo2.70131)
Supplement: Supplementary file 1 [file IMO2-3-e70131-s001.docx]

# **Supporting information to**

# Climate change amplifies global mycotoxin exposure: Emerging risks and sustainable solutions

## **Running title:** Mycotoxin risks to global food security

Mengyao Xue^1^, Zheng Qu^1^, Tingting Jiang^1^, Antonio F. Logrieco^2^, Antonio Moretti^3^, Ke Dong^4^, Yanpo Yao^1*^, Qi Zhang^5*^, Peiwu Li^5*^

^1^Agro-Environmental Protection Institute, Ministry of Agriculture and Rural Affairs,

Tianjin 300191, China

^2^Xianghu Lab, Biomanufacturing Institute, Hangzhou 311231, China

^3^Institute of Sciences of Food Production, National Research Council, Bari 70126, Italy

^4^Kyonggi University, South Korea, Seoul 100-744, South Korea

^5^Key Laboratory of Detection for Mycotoxins, Ministry of Agriculture and Rural Affairs, Oil Crops Research Institute, Chinese Academy of Agricultural Sciences, Wuhan 430062, China.

# Correspondence: [yaoyanpo@caas.cn](mailto:yaoyanpo@caas.cn) (Yanpo Yao); [zhangqi01@caas.cn](mailto:(zhangqi01@caas.cn);) (Qi Zhang); [lipeiwu@caas.cn](mailto:(lipeiwu@caas.cn)) (Peiwu Li)

**Figure S1 Schematic overview of the four priority actions proposed to mitigate climate-driven mycotoxin contamination.** The measures are systematically arranged in a coherent progression, beginning with early warning, followed by detection and source-level prevention, and culminating in global governance. Collectively, these measures constitute a comprehensive framework aimed at protecting food security and public health in the context of climate change.

**MATERIAL AND METHODS**

#### **Data sources for mycotoxin prevalence**

Mycotoxin prevalence data used in this commentary were derived from three complementary sources. First, we systematically extracted published prevalence data from peer-reviewed literature identified through our literature search. Second, we incorporated official industry survey reports, including Cargill Global Mycotoxin Reports and DSM-Firmenich World Mycotoxin Surveys. Third, to complement these existing datasets, we included unpublished results from our own large-scale field monitoring program conducted by the Agro-Environmental Protection Institute, Ministry of Agriculture and Rural Affairs. The program collected maize, wheat, and rice samples from the major grain-producing regions of China during the 2021-2025 growing seasons. For all three data sources, we extracted contamination rates and where reported, mean mycotoxin concentrations.

**Literature search and selection criteria**

#### Given the narrative review nature of this commentary, we performed a targeted literature search in PubMed, Web of Science, and Scopus using combinations of the following keywords, mycotoxin, climate change, global warming, aflatoxin, deoxynivalenol, fumonisin, food security, biosensor, and predictive model. The search covered publications from 1980 to 2025. Additional relevant studies were identified through citation snowballing from key reviews and the reference lists of included articles. We prioritized peer-reviewed original research, systematic reviews, and official reports from international organizations.

#### **Mycotoxin detection by liquid chromatography-tandem mass spectrometry (LC-MS/MS)**

All mycotoxin concentrations in our own field samples were determined using LC-MS/MS. Sample preparation followed a standardized protocol involving acetonitrile/water (80:20, v/v) extraction, followed by clean-up using QuEChERS d-SPE. Chromatographic separation was achieved on a C18 column (2.1 × 100 mm, 1.7 µm) with a mobile phase gradient of methanol and 0.1% formic acid in water. The mass spectrometer was operated in multiple reaction monitoring (MRM) mode with electrospray ionization (ESI). The method was validated for linearity (R² > 0.999), recovery (92.5-97.2%), and repeatability (relative standard deviation < 15%). Limits of detection (LOD) and quantification (LOQ) ranged from 0.1-1.0 μg/kg depending on the mycotoxin.

**Climate and mycotoxin modeling framework**

To project future contamination risks, we employed the AFLA-maize mechanistic model, which simulates the infection cycle of *Aspergillus flavus* on maize as a function of daily temperature, water activity, and crop phenology [1]. The model outputs the probability of aflatoxin B1 (AFB1) concentrations exceeding the EU regulatory limit at harvest. Meteorological inputs for the model were derived from the Coupled Model Intercomparison Project Phase 6 (CMIP6) under two Representative Concentration Pathways: RCP 8.5 (high-emission scenario, ~1370 ppm CO_2_-equivalent by 2100) and RCP 2.6 (low-emission scenario, peaking at ~490 ppm CO_2_-equivalent before 2100 and declining thereafter) [2]. The baseline historical period was defined as 1980–2010, while future projections were assessed for the mid-century period 2040–2070. The relative changes (%) in contamination ratio presented in Figure 2 were calculated as the percentage difference between future projections and baseline outputs for each region.

**Supporting tables and figures**

Table S1 summarizes the optimal temperature and water activity conditions for mycotoxin production as reported in the cited literature. Table S2 compares major mycotoxin detection methods based on a systematic evaluation of published studies identified through our literature search, categorized by detection principle. Figure S1 was constructed based on the four priority actions synthesized from the literature reviewed and the authors’ expert consensus on climate-driven mycotoxin risk mitigation.

#### **Data availability**

#### Due to legal, institutional, and confidentiality restrictions, the mycotoxin and meteorological data generated by the Agro-Environmental Protection Institute are not publicly available. Requests for access should be directed to the corresponding authors as detailed in the Data Availability Statement in the main text.

**REFERENCE**

1. Warnatzsch, Erika A., David S. Reay, Marco Camardo Leggieri, Paola Battilani. 2020. “Climate Change Impact on Aflatoxin Contamination Risk in Malawi's Maize Crops.” *[Frontiers in Sustainable Food Systems](https://www.frontiersin.org/journals/sustainable-food-systems)* 4: 591792. <https://doi.org/10.3389/fsufs.2020.591792>
2. IPCC. 2021. “Climate Change 2021: The Physical Science Basis. Contribution of Working Group I to the Sixth Assessment Report of the Intergovernmental Panel on Climate Change.” Cambridge University Press.
